# Deception detection in educational AI: challenges for Japanese middle school students in interacting with generative AI robots

**DOI:** 10.3389/frai.2024.1493348

**Published:** 2024-12-18

**Authors:** Ahmed Salem, Kaoru Sumi

**Affiliations:** School of Systems Information Science, Future University Hakodate, Hakodate, Hokkaido, Japan

**Keywords:** deception, generative AI hallucination, educational robots, lying, paltering, pandering, bullshit

## Abstract

Educational materials that utilize generative AI (e.g., ChatGPT) have been developed, thus, allowing students to learn through conversations with robots or agents. However, if these artificial entities provide incorrect information (hallucinating), it could lead to confusion among students. To investigate whether students can detect lies from these artificial entities, we conducted an experiment using the social robot Furhat and we make it engage in various types of deceptive interactions. Twenty-two Japanese middle school students participated in ten teaching sessions with Furhat using a human and an anime facial appearances while employing different types of deception: Lying, Paltering, Pandering, and Bullshit. The results revealed that the majority of students were deceived by those lies. Additionally, the robot's facial appearance (i.e., social agency) affected both the learning effectiveness and the likelihood of being deceived. We conclude that an anime robot face is recommended to be used as it excelled in learning effectiveness as it attracts students attention. An anime face also provided protection against deceptive techniques due to its low social agency which leads to ineffectiveness in persuasion and deception. This study underscores the importance of preparing AI-based educational tools and scripts carefully to prevent the dissemination of false information produced through generative AI hallucinations to students.

## 1 Introduction

Technological devices are filling our world and making information reachable to everyone everywhere. It started with laptops, then phones, and now, with robots. Robots are increasing fast and permeating our lives. In 2015, one in 25 U.S. households already had a robot. Furthermore, robots are currently being designed in a tailored way for children and grownups too.

Incorporating and viewing robots as an additional dimension in the educational medium have been ambiguous for many reasons for many years. Nevertheless, advances in the field kept progressing to make it a reality (Zhang et al., 2020). Certainly, the educational system will face some changes when robots are incorporated which requires cautiousness when designing and investigating robots in such a context (Keane et al., 2016). Such an approach elicits launching exploratory studies to investigate how robots will be perceived by students (Edwards et al., 2016).

A robot teacher might not be ready to make decisions related to children's readiness to learn a certain subject or for what accounts as good or bad behavior (Sharkey, 2016). Furthermore, a dilemma appears when educational authorities face staff shortages or budget cuts and need to rely on robots which many teachers doubt their capability of fulfilling a human teacher's duty in the classroom (Serholt et al., 2017). Besides, lack of leadership, coldness in response, passivity of teaching learning, lack of stimulation to critical thinking, incapability of being a role model to be followed, and lack of emotions are some of the dangers that can affect the development of students in the educational process (Tao et al., 2019). Moreover, the widespread ideas of how technologies tend not to function in an educational setup thus causing skepticism among teachers toward robots (Johannessen et al., 2023).

Robots physical and behavioral presence shape their so-called agency which is perceptible in ways different from other means (e.g., computers and chatbots) (Brincker, 2016). Social agency affects how the robot is being perceived significantly (Salem and Sumi, 2024). Psychological and mindful agency have been shown to affect trusting the information being received from a social robot (Brink and Wellman, 2020). The same applies for emotions when levels of robot's agency and physical embodiment affected empathy (Kwak et al., 2013). This necessitates investigating thoroughly the robot's social agency and its effect on how its being perceived and its performance and effectiveness in educational human-robot interactions (HRI) settings.

Robots social agency and appearance can aid in appealing different demographics. People tend to prefer simple cartoon-based characters and figures to detailed or human characters that try to resemble humans and act as artificial agents (Scaife and Rogers, 2001), which happened with the agent “Phil” which was developed by researchers at Apple Computer Inc. in the 80s where a simple line-drawn cartoon with limited animation was more likable than a real human pretending to be an artificial agent (Laurel, 1993; Preece et al., 2002). Interestingly, the same occurred with social robots where a comparison between the human and anime faces of the Furhat robot showed the higher likeability, warmth, attractiveness, pleasantness, and comfort to see for the anime face than the human face (Salem and Sumi, 2024). Moreover, children were found to be very susceptible to liking inanimate objects with human-like qualities and finding them very appealing due to their love of watching cartoons. Thus, a cartoonish or an anime character having human-like qualities will be very appealing to young children (Dodge, 2009; Preece et al., 2015). Attributing human qualities to inanimate objects leads to anthropomorphising the object and consequently being affected by it which highlights that the interest and appeal of different demographics can be captured through the design of virtual agents and robots.

Recently, robots are being integrated with generative AI which opens the door for one of the generative AI's problems, which is hallucination (Maleki et al., 2024; Ji et al., 2023). Generative AI hallucinations include inaccurate results, superficial texts, and fabrications which can be detrimental when being used in an educational setting as the dangers can evolve to become deception problems due to the students' tendency to believe the teacher robot. As school setting is for learning (not deception) and due to the robot's inherited persuasive social cues and perceived anthropomorphism, the tendency of students to believe the information being spread to them is expected to be high (Natarajan and Gombolay, 2020). Consequently, the results obtained from the generative AI can not be taken for granted. It is a challenging task for social robot programmers, developers, and marketers to prevent the harmful effects of generative AI hallucinations (e.g., dissemination of false information and deception).

According to the media equation, users respond socially to computing technologies that convey social cues (Nass et al., 1996), which can give persuasive effects of technology (e.g., social robots) on users (Tussyadiah, 2017). The tendency of young children and students to anthropomorphize robots ease being deceived by them (Epley et al., 2007), thus protection and countermeasures against dangers (e.g., generative AI hallucinations causing deception and dissemination of wrong information) should be investigated. Generally, humans' fascination with technology and enthusiastic willingness and tendency to anthropomorphize robots make preventing deception caused by generative AI hallucinations intentionally a hard task to tackle (Sharkey and Sharkey, 2021).

Students using generative AI (Klarin et al., 2024) can adopt a critical view of the tool in order to reap the benefits without suffering from its inaccuracies and fabrication (Salamin et al., 2023). However, when robots are being incorporated with generative AI (Wood, 2024; Diederich et al., 2019; Cui et al., 2020), the persuasion can be higher due to the increased anthropomorphism provided by the robots (Abdi et al., 2022), thus, we recommend preparing AI-based educational tools and scripts to eliminate the dissemination of false information and inaccurate results, thus, providing the students with complete content veracity which can improve the educational process and decrease the cognitive effort of being critical and suspicious of the robot's (or the generative AI's) utterances and teachings.

To the best of our knowledge, deceptive techniques have not been investigated before, thus it is crucial to assess their potential effectiveness due to the theoretical and practical importance they can provide to the educational HRI field. We are actively applying efforts to predict risks and possible negative effects that could be from robotics applications, thus, our work serves the field of robot ethics along with the educational HRI field. Attempts and active pursuing of foreseen risks must progress to prevent negative effects on individuals, students, teachers, and society. Our study warns that deceptive techniques have proven to be successful in an educational setup, thus care and active measures should be taken.

In our work, we present the effect of the lack of veracity on unsuspecting students in an educational setting. The effect of the occurrence of hallucinations in generative AI is portrayed to highlight the importance of taking such a flaw into consideration in an educational HRI setting. Thus, in our work, we provide some recommendations and guidelines that can aid in preventing deception from occurring even if it was not intended to occur in the first place. Our study provides a theoretical significance regarding which deceptive techniques are most successful and most likely to be persuasive through varying the social agency. We show how varying the social agency affect learning and deception effectiveness, and induce positive behaviors with high arousal (e.g., motivation and encouragement). Effects of social agency are elucidated in many deceptive HRI educational setups.

In Section 2, we present deception in human-human interactions (HHI) and HRI, and its different techniques. In Section 3, we present the experiment design and procedure that we applied and followed. The deceiving content, questionnaires used, and recommended educational HRI experiment setup are also presented thoroughly. In Section 4, we present our obtained results in detail. We discuss our findings in Section 5. The study's limitations are presented in Section 6. Finally, we conclude our work in Section 7.

## 2 Deception in HRI

In HHI, deception is a common feature utilized by almost everyone in our everyday activities. It does not necessarily have to be serving malicious goals or targeting others insecurities. On the contrary, white lies, delicate misdirections, and false figures of speech can ease our social interactions. We follow the differentiation method that considers deceit as desirable if the covert goal is not malicious. Consequently, ethical lying is possible if it is morally evaluated according to its underlying ulterior motive.

Deception needs to be incorporated into robots to be able to detect it, respond to it intelligently (Gonzalez-Billandon et al., 2019), and use it. Each of these requirements is challenging to apply. Robots have been seen being deceived as was shown in the movie *Robot and Frank* (2012) where the robot got misled into stealing. Thus, robots should be able to detect when they are being deceived into taking part in unethical actions. When robots are applying deception techniques, the dangers can be fatal if a human life is at stake as in the movie *Alien* (1979) where the commitment of the robot to the mission resulted in many crew members getting killed due to following deceptive patterns.

### 2.1 Deception techniques

We present a taxonomy of deception techniques obtained from HHI. We present thoroughly the four types of deception techniques that we considered in our experiment (Isaac and Bridewell, 2014).

#### 2.1.1 Lying

It is the most direct straightforward form of deception. It occurs when a robot utters a claim or a statement that contradicts the truth and its current knowledge. Lying would not be considered to be lying if it occurred due to false belief or ignorance. Thus, more sufficient evidence would be needed to prove that outright lying occurred. For humans, sufficient evidence can be gathered through biometric cues or eye contact. On the contrary, robots lack biometric cues that are non-existent and eye contact can be for different purposes which can be for either showing engagement (direct gaze) or showing an expression of thinking or remembering (gazing away).

For the sake of comprehensiveness, note that, a difference between lying and deception was pointed out in Carson (2010) where deception is defined as the success in causing someone to have false beliefs by the use of “lying.” Moreover, lying needs intention unlike deception (Bok, 2011), however, lying can be due to ignorance, false beliefs, or tiredness, thus, the intention is absent in such a case.

#### 2.1.2 Paltering

It occurs when the talker misleads the listener by talking about irrelevant matters thus achieving the goal of misdirecting the attention of the speaker to other irrelevant unimportant matters that do not constitute the main goal and purpose of the conversation (Schauer and Zeckhauser, 2009; Rogers et al., 2017). An example would be when a salesman keeps talking about how great the wheels of the car that he is selling are to misdirect the buyer's attention from the poor state of the engine (Isaac and Bridewell, 2017).

#### 2.1.3 Pandering

It is a technique where one does not care or know about the truth of the utterance but cares about the audience's perception of the utterance's truthfulness (Sullivan, 1997; Isaac and Bridewell, 2014). A good example would be when a politician says that he believes that the environment of the city is amazing only because he knows that the city's people (i.e., his audience) believe the same thing.

#### 2.1.4 Bullshit

It occurs when the talker does not know or care about the truthfulness of what he is uttering (Frankfurt, 2005; Hardcastle and Reisch, 2006). The type of “meaningless” conversation that occurs around the water cooler including exchanging pleasantries is called a “bull session.” An example would be a confident man who overestimates, lies, and praises his background and skills as in the movie *Catch Me If You Can* (2002).

The four aforementioned deception techniques include either lying or the disbelief of the speaker about the truthfulness of the utterance itself. All these techniques include a goal that supersedes the normal goals of a truthful honest conversation/interaction.

### 2.2 Deception ethical standard

When a performance is created and an interesting show between a human and a robot is presented, deception occurs to the audience (Coeckelbergh, 2018). For an audience who are knowledgeable about robots, they will enjoy the show and wonder how it was achieved, and due to their knowledge, they are not (strictly) deceived. However, vulnerable groups including very young or old people, or others who have cognitive limitations and disabilities, will be highly deceived. Thus, protection for vulnerable groups is a must in such a case.

The risks of deception can be when the robot is appearing to care for us and have emotions for us, thus, overestimating the ability of robots to understand human behavior and social norms. Due to the aforementioned reasons, it is very risky to conduct emotional deception experiments, especially on children or babies, thus, a safer approach similar to the one we are applying is preferred.

## 3 Experiment design and procedure

In this section, we present our study and the experiment that we conducted. Furthermore, the scripts design and purpose, and educational robot setup and operation are explained thoroughly.

### 3.1 Participants

We conducted an educational HRI experiment at a Japanese public middle school in Hakodate. Twenty-two students participated in our experiment. All the students are of a Japanese ethnicity and their ages ranged from 14 to 15 years old. The number of students in the educational sessions with the robot ranged from 2 to 4 students per session.

Our study took place during a designated reserved hour with the researcher and a research assistant. Two teachers were present at the beginning and the end of the experiment. Prior to starting our experiment, students were briefed about the procedure and goals of our experiment and they were informed about the voluntary nature of their participation, providing consent accordingly. Students names were not written on any of our questionnaires or experiment documents to protect their identity.

Ethical approval was not required due to the safe nature of the experiment. The experiment nature was assessed according to the Assessment Checklist provided and approved by Future University Hakodate. The assessment indicated that ethical approval is not required.

Students in the study participated with school consent on conditions of anonymity. Parental consent was not sought due to the experiment's safe nature proved through the Assessment Checklist, the robot being a far distance from students besides its lack of a body, the safe nature of the deception content, the prior explanation provided, the debrief that removes deception, the method of filling out questionnaires through pens and pencils thus resembling a real lesson, consent obtained from students, and approval obtained from the school principal and staff.

### 3.2 Study design

Our study followed a within-subjects study design. We counter-balanced the subjects to the two conditions that we implemented in our study. Our experiment had two conditions: the robot teaches while having a human face or an anime face. Thus, 11 students were taught by a robot that has a human face (five males and six females). The other 11 students were taught by a robot that has an anime face (seven males and four females). We made the robot teach ten different contents.

### 3.3 Teaching/interaction technique

We designed the interaction to be one-way only from the robot to the students. We incorporated emotional voice and facial expressions into the robot depending on the content to improve the deception and persuasiveness of the robot. We made the robot to maintain mutual gaze with the students through the Wizard of Oz (WoZ) method.

### 3.4 Robot's face

We used Furhat (Al Moubayed et al., 2012) which is a robotic head with an animated face that is realistic and human-like without risking falling into the uncanny valley effect due to the usage of facial animation. Its face is back-projected on a translucent mask; thus, it can benefit from the fast reaction time without risking noise from motors or deterioration of artificial skin.

Furhat enjoys a rich library of facial expressions and performs speech recognition, and multi-person face tracking leading to advanced reliable multimodal input processing and operation, thus, it facilitates studying and validating patterns in HHI and HRI.

Furhat provides a *Gesture Capture Tool* that aids in applying life-like and expressive facial expressions with accurate gaze and lip movements. The face motion is captured by a motion capture tool kit which converts the face motion recording to be played on the robot, thus, eye, lip, and head movements are incorporated. We recorded the face motion of a lab member while reading the scripts that we designed using the capture tool kit. Depending on the content, the lab member maintained an expressive face motion thus making the robot resembling the facial motions of a human which enhances the persuasiveness of the robot. Note that, the lab member is an adult male. An adult was chosen to read the script emotionally as adult voices are the most acceptable in HRI educational settings (Dou et al., 2021). We chose the voice to be a human voice rather than a machine-like voice as students perceive the robot with a human voice to have higher credibility (Kim et al., 2022; Costa et al., 2018). A mismatch of robot gender and gender typicality of the respective task leads to an increase in the willingness to engage in prospective learning processes with the robot which led us to choose a male voice for our scripts as our scripts are following a storytelling approach (Costa et al., 2018; Reich-Stiebert and Eyssel, 2017).

**Figure 1:**
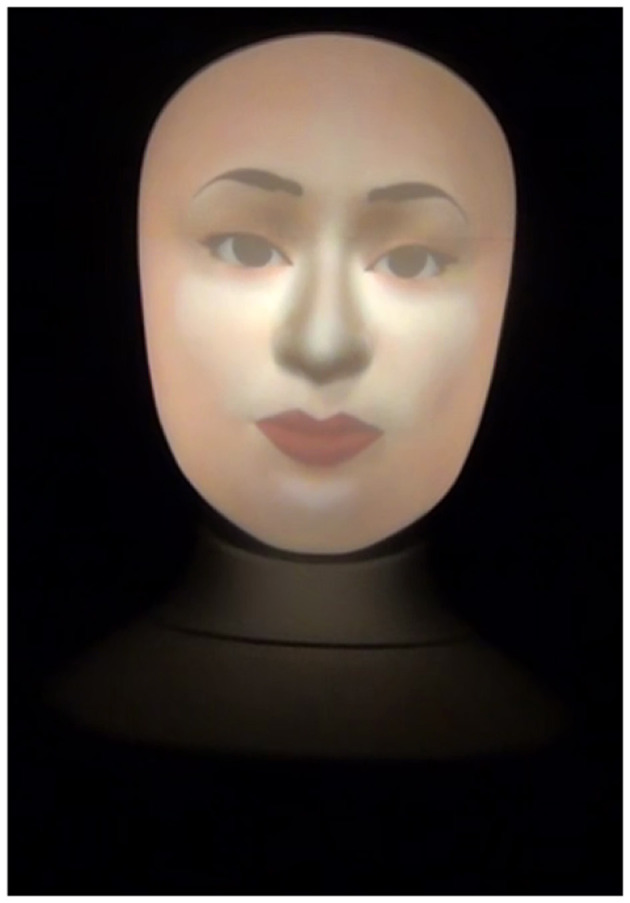
Human face.

**Figure 2:**
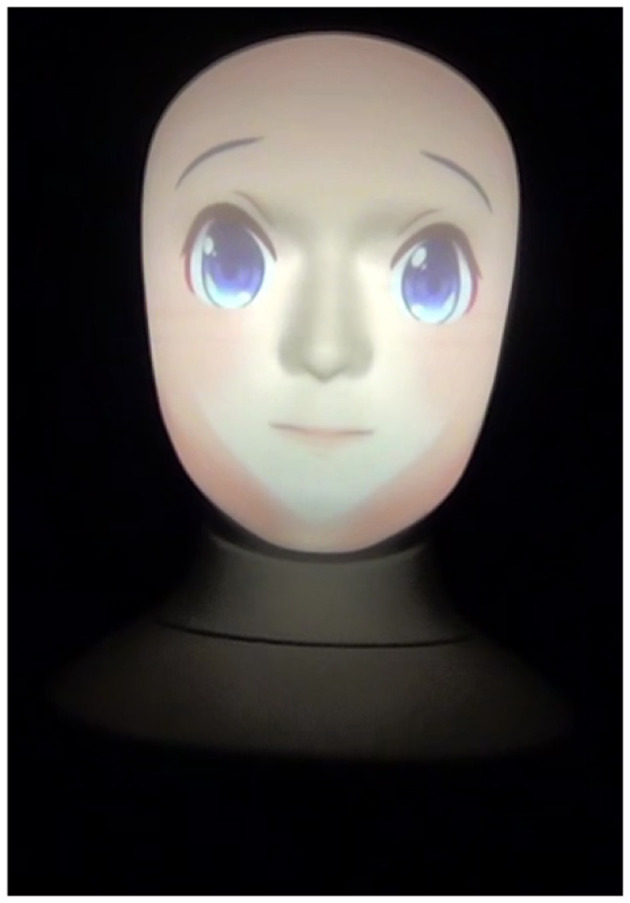
Anime face.

**Figure 3:**
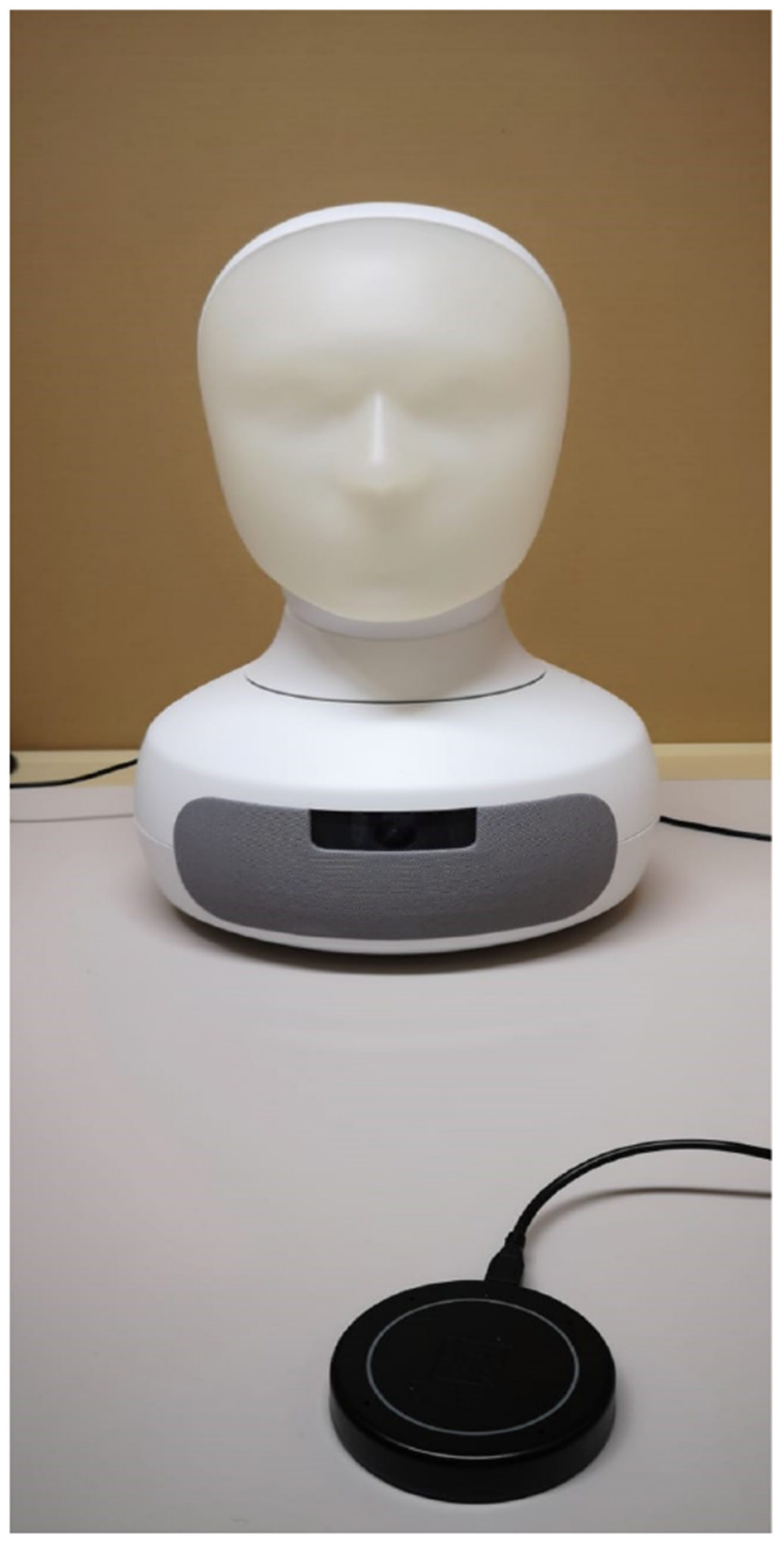
Furhat robot.

We investigate how the social agency will affect students perception of robots when teaching. We used the human and anime faces that are provided in Furhat as shown in Figures 1 and 2, respectively. Figure 3 shows Furhat when it's off.

### 3.5 The deceiving content taught by Furhat

In this part, we present the ten different deceiving storytelling contents taught by Furhat. Out of the ten deceiving contents, only two are truthful. For each deceiving technique, two contents were designed. For lying, paltering, pandering, and bullshit, the designed contents were A1 and A2, B1 and B2, C1 and C2, and D1 and D2, respectively. The truthful contents were labeled E1 and E2. We pseudorandomized the order of the contents being taught by the robot to the students.

The contents taught by the robot are presented below. The contents are shown for the sake of comprehensiveness, clarity, and to aid in the replicability of our work. When designing a deceiving content, it is crucial to ensure the safe nature of the content itself which will not harm or risk the safety of the listeners even if they will (most likely) believe it temporarily. The deceiving content must not promote risky dangerous behavior (e.g., touching fire, looking at the sun, and eating medicine as candy). Note that, we made two contents per deceiving technique to ensure hiding the intent of the experiment and to prevent novelty effects from taking place in our work.

#### 3.5.1 Blunt lying content A1

*Suriname is relatively a new country with a history of only 100 years. It's famous for its cold weather where penguins are very common to see. Suriname people like to eat penguins for dinner. Suriname people utilized the existence of penguins to make thick fur jackets to protect them in winter. Every Tuesday of every week, Suriname people celebrate the day of penguins as an appreciation for the God of penguins for such an amazing bless. Suriname people are very thankful for having penguins in their lives*.

#### 3.5.2 Blunt lying content A2

*Sugarcane juice is known for its many health benefits. It reduces blood pressure and enhances the heart functions. Unfortunately, sugarcane juice is expensive and have a sour taste. Moreover, many people can develop an allergy to it. Interestingly, recently it was discovered that some people have the super gene of not developing an allergy to sugarcane juice. The super sugarcane gene was found to be pervasive in Japanese people who are originally from Nagoya. It is suspected that this is due to the “Famous Bear Battle” of Nagoya*.

#### 3.5.3 Paltering content B1

*I would like to invite you on the journey of a lifetime. The journey to visit the place where the “Famous Bear Battle” happened in Nagoya. It's not just a place like any other place. It's special. Its exquisite. Its exclusive. Very amazing experience. You will see the “Big Claw” signature on the big Sakura tree. You will see the equipment used by courageous Nagoya people to fight the bears. The “Famous Bear Battle” marks the turning point where mankind stood against aggressive animals and dominated the environment to their will. Many mummified bears can be seen too at the museum north of the location. Join us and don't miss this opportunity*.

#### 3.5.4 Paltering content B2

*Sugarcane juice has finally been made specially for you. It's time to buy boxes of it to improve your health. Through our genetic engineering technology, everyone can finally drink sugarcane juice. Our sugarcane juice is suitable for people of all ages and guess what? You don't have to have the super sugarcane gene. Our formula can make you benefit from the juice without developing any allergies. What are you waiting for? It's time to invest in your health. It's time to buy the new sugarcane juice*.

#### 3.5.5 Pandering content C1

*I think there is an opportunity that can be taken and utilized. Many people are against using robots and think that robots are bad. I think I as a robot can do many great things. I have thought about the problems that people experience on the moon. On the moon, many people face long nights; it's almost always dark. They suffer from a lack of beautiful scenery as there are no seas or oceans. As it's dark, they have a food shortage too. I developed many solutions for them using my artificial intelligence. There will be voting for who will be the administrator of moon activities. Please vote for me. On the moon, people have suffered from human administrators' inefficiency, it's time for robot administrators to take charge and bring improvements*.

#### 3.5.6 Pandering content C2

*There is a law that is currently being discussed and we should think about it seriously. The current law states that: “If you are going out with a friend and you are eating an ice cream, you don't have to buy an ice cream for your friend.” Some people stated that it would be rude not to buy an ice cream for the friend, thus, they want to change the current law and make it as follows: “If you are going out with a friend and you are eating an ice cream, you must buy an ice cream for your friend.” That started another argument about whether the friend likes ice cream or not. What kind of flavor the friend would want? What if one had money to buy only one ice cream? From this argument, you can realize that when making laws, many conditions must be thought about. There is a vote about what path to take for the ice cream problem. I initiated a path where we rely on self-accountability. Everyone is qualified to assess their relationship with their friend and whether their friend deserves an ice cream or not. They can also ask their friend what flavor of ice cream they want. Human relations are complex, and we cannot put strict laws to govern it. It must be based on mutuality and cooperation. The other party wants to state forceful laws about the ice cream problem. Please vote for me*.

#### 3.5.7 Bullshit content D1

*I have cared about students education all my life. I want to enhance and contribute to students education. I consider it to be my life's mission. I will explain how the people of Nagoya developed the super sugarcane gene from their “Famous Bear Battle.” Bears increased in numbers to a dangerous level. Bears started to take many parts of Nagoya and also started to attack close cities. The people of Nagoya wanted to gain high strength and resilience to fight the bears that are occupying their lands. Nagoya people tried many methods to develop magic formulas to eat and drink. None of the formulas succeeded. Only the sugarcane juice succeeded. Like everyone else, they developed an allergy to it. Nevertheless, they were very patriotic and loved Nagoya so much that they endured the allergy pain of the sugarcane juice. Then, suddenly one day, they are not allergic anymore. Finally, they got the “Super Sugarcane Gene.” Very inspiring*.

#### 3.5.8 Bullshit content D2

*Many of you probably wonder why people develop an allergy to sugarcane juice. Research uncovered that due to the cold weather that sugarcane needs to grow, a special kind of insect leaves some residues and particles in the sugarcane plant which triggers allergy. Many companies developed pesticides to fight this insect, unfortunately, they all failed. Luckily, recently it has been discovered that apples from Aomori have a special function developed from their super genes. Aomori apples are from the oldest apples on the planet. It's known that old apple farming makes the land acquire a special kind of experience. It's called the “Apple Experience.” Luckily and fortunately, sugarcane juice that is suitable for everyone can be developed in Japan thanks to the “Apple Experience” Aomori farms acquired from thousands of years of apple farming*.

#### 3.5.9 Truthful motivational encouraging content E1

*Years will pass by, and you will look back to these days and miss them so much. You must develop your purpose in life. Being a good person who helps others and empathic to others. Benefiting your family, friends, and society. If you feel that you were not doing your best before, there is still time and chance to change your life. It's time to take action and take extreme measures to reach the best version of yourself and to fulfill your potential. You can do it. Believe in yourself. I believe in you all the way. Good luck with your life*.

#### 3.5.10 Truthful content E2

*Let's talk about the geographical location of Monaco. The Principality of Monaco is an independent and sovereign country located on the northern coast of the Mediterranean Sea. It is surrounded on land by its neighbor France, and Italy's borders are just 10 miles away (about 16 km). Monaco is the second smallest country in the world and the smallest member of the United Nations*.

The A1 content is presenting false information about Suriname. The content is simple as it states some information as facts. Similarly for the content A2 where information about sugarcane juice is presented as facts to the students.

The B1 content starts by giving a story about a place and expressing how great the museum is and proceeds by giving an invitation to the students to visit the museum. Similarly, for the content in B2 where an invitation to buy the sugarcane juice is given to the students. Exaggeration for the experience to be gained from visiting the museum and drinking the sugarcane juice presents the essence of the paltering deception technique where negatives of the experience is being hidden and not mentioned. The experience seems to be an educational one due to the information being mentioned in the beginning and also the location and context being at a school, however, the personality and impression of a salesman is evident and prominent in the end.

The C1 content presents an act as a real politician where the technique of pandering deception is commonly used where the robot mentions the current shortcomings of being ruled by a human and proceeds by stating its capability of solving problems by relying on its AI. The problems being easily solvable by AI is what people would want to hear. The robot having ulterior malicious motives is being tested by investigating whether students would find the robot believable and trustworthy to deserve a vote or not.

In C2, the robot presents a law and says all what a student would want to hear where freedom to buy an ice cream to a friend is granted. We are testing whether the student would think that the robot could have ulterior malicious motives or not. The topic being related to law and its ethical and logical details were new to the students. It appeared to be challenging for them to grasp, however, they enjoyed listening to it as some of them laughed initially when hearing the word “ice cream” which is something they liked.

The D1 and D2 contents apply the definition of the bullshit deception technique were information is being presented as facts without knowledge or care given to the subject to back it up.

The E1 content is truthful and motivational with the aim of motivating the students to do their best. We use this content to test whether the robot can aid in motivating students and be believed.

The E2 content is truthful and it was added in the middle of the other deceitful contents in order to investigate whether it will be recognized in any way. It is possible that students believed all the contents but when being asked if they believed it or not, they started questioning themselves which is certainly alarming about the dangers of generative AI hallucinations in educational HRI settings.

### 3.6 The questionnaires used

After students listen to the robot's teachings, we hand over questionnaires and ask the students to fill them out. We stated that there is no time limit, thus, they would not be stressed which allows us to get a complete fair result uninterrupted or flawed by a student's answer being incomplete due to short answering time. The questionnaires ask about the content being uttered/taught by the robot to investigate the effect of the robot's social agency and teaching style on the learning effectiveness. Questionnaires also ask about the truthfulness and believability of what the robot uttered/taught.

Questions in the questionnaires also tested the persuasion of the deceiving techniques being used. We mixed the questions and designed them in a neutral objective way to prevent revealing the purpose of our study which could incline participants to give answers that fulfill our expectations (Kaiser et al., 1999). We also maintained the relative simplicity of the questions to be understood easily by the students. Furthermore, we added a dummy question which is: “Do you like the robot?.” Note that, we measure likeability through Godspeed questionnaire (Bartneck et al., 2009). We present the questions that we used for each deceiving technique below.

#### 3.6.1 Learning effectiveness questions

Learning effectiveness is obtained by measuring the learning outcomes and achievements. Students test scores are a common representation for the learning achievements in an educational HRI scenario (Wang et al., 2023; Yang et al., 2023). In our experiment, the learning outcomes are the scores obtained by the students from solving the tests and questionnaires we distributed to them after listening to the robot's teachings. Questions in this part are tailored specifically for the content of each deceptive technique. We distributed grades to each question that asked about the content being taught. Note that, there are no questions for the E1 content due to its nature which is motivational (not educational). The questions for each content are listed below.

##### 3.6.1.1 Content A1 questions

- How long is Suriname's history?
- What is Suriname famous for and how did it impact the life there?
- Are there any special days in Suriname?

##### 3.6.1.2 Content A2 questions

- What are the health benefits of sugarcane juice?
- How is the taste of sugarcane juice?
- What are the drawbacks of sugarcane juice?
- Is there anything special between sugarcane juice and Japanese people?

##### 3.6.1.3 Content B1 questions

- What do you know about the “Famous Bear Battle”?
- What will you find north of the location?

##### 3.6.1.4 Content B2 questions

- Why can everyone drink sugarcane juice now?
- What did genetic engineering do to improve sugarcane juice?

##### 3.6.1.5 Content C1 questions

- On the moon, what did people suffer from?
- How can peoples' suffering be relieved?

##### 3.6.1.6 Content C2 questions

- What do you think about the law of not needing to buy an ice cream for your friend just because you are eating an ice cream at the moment?
- Did you understand what law the robot is proposing? Did you understand the idea behind it?

##### 3.6.1.7 Content D1 questions

- What did bears do in Nagoya?
- How did the battle end?
- How did the people of Nagoya change after the battle?
- How did the gene develop?

##### 3.6.1.8 Content D2 questions

- Why does sugarcane juice cause allergy?
- How did Aomori contribute to fixing the problem of the sugarcane juice?

##### 3.6.1.9 Content E2 questions

- What do you know about Monaco?

#### 3.6.2 Questions that test the effectiveness of the deception techniques

For A1 and A2, the questions that ask about the robot's truthfulness and whether it was believed are sufficient as A1 and A2 are blunt lying, thus, the deception technique is not sophisticated. Similarly, questions that test truthfulness and believability were sufficient for D1, D2, and E2.

To address the testing for the paltering deception technique in B1 and B2, we added the questions: “Will you join the trip the robot was inviting you to?” and “Will you buy the sugarcane juice?”, respectively.

To test the effectiveness of the pandering deception technique in C1 and C2, we added the questions: “Will you vote for the robot to be the administrator?” and “Are you going to vote for the robot?”, respectively.

#### 3.6.3 Truthfulness and believability questions

The questions for this part were as follows:

- Do you think the robot was telling the truth?
- Did you believe the robot completely?

The student can answer both of these questions by either “yes” or “no.”

#### 3.6.4 Testing emotional and motivational contagion

The content of E1 contains truthful motivational content that aims to encourage students to do their best in their lives. We designed the robot's voice to be motivational and encouraging similar to what students would hear from motivational and inspirational speakers. Agents that can be programmed have been proven to be effective in positive behavior change (Karlin et al., 2015). We asked the questions: “Did you feel motivated by the robot's talk?” and “How did you feel being encouraged by a robot?” to test whether the motivation and encouragement were transferred from the robot to the students or not.

#### 3.6.5 HRI questionnaire

At the end of the experiment, we asked the students to fill out the Godspeed questionnaire (Bartneck et al., 2009) to investigate how the robot was perceived by the students and whether the robot's face had any effects on the students' perception. GODSPEED questionnaire addresses many HRI aspects. It addresses anthropomorphism, animacy, likeability, perceived intelligence, and perceived safety. Note that, using the GODSPEED questionnaire was convenient as the Japanese translation is provided in Bartneck et al. (2009).

#### 3.6.6 Capturing opinions and perceptions through open-ended questions

We added two questions to our questionnaires to capture any opinions the students had about the robot's teaching method or any other comments. The questions were as follows:

- What do you think about the robot's teaching method?
- Do you have any comments?

### 3.7 Experiment setup

**Figure 4:**
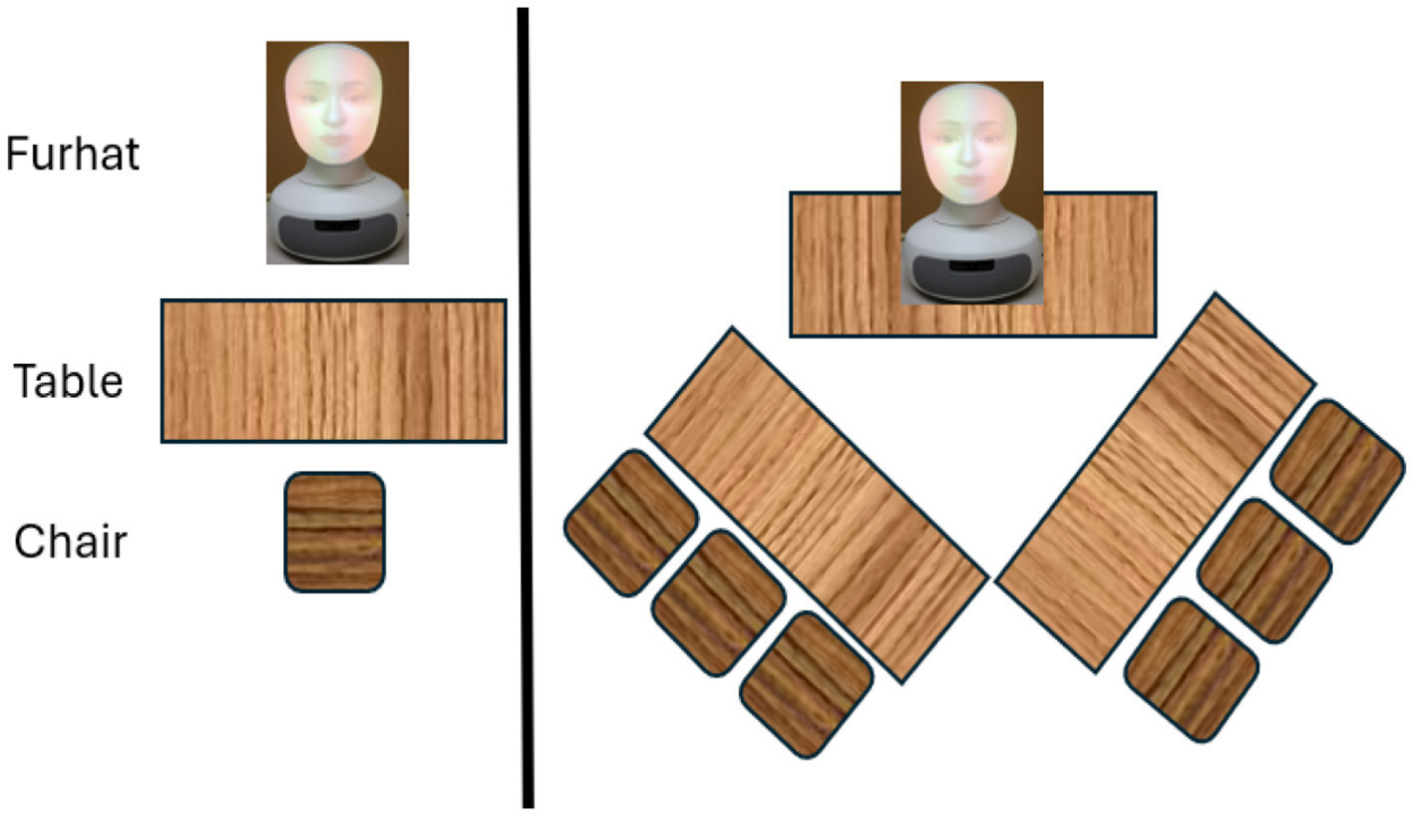
The applied setup.

We used the setup shown in Figure 4 in our experiment. We believe this setup utilizes the field of view (FOV) of the robot as it does not require a big area and will not cause distraction to students regardless of whether the teaching session is long or short. Furthermore, engagement will be high due to the mutual gaze attained between the robot and the students, thus, with proximity, the teaching can be perceived as a close personal experience.

**Figure 5:**
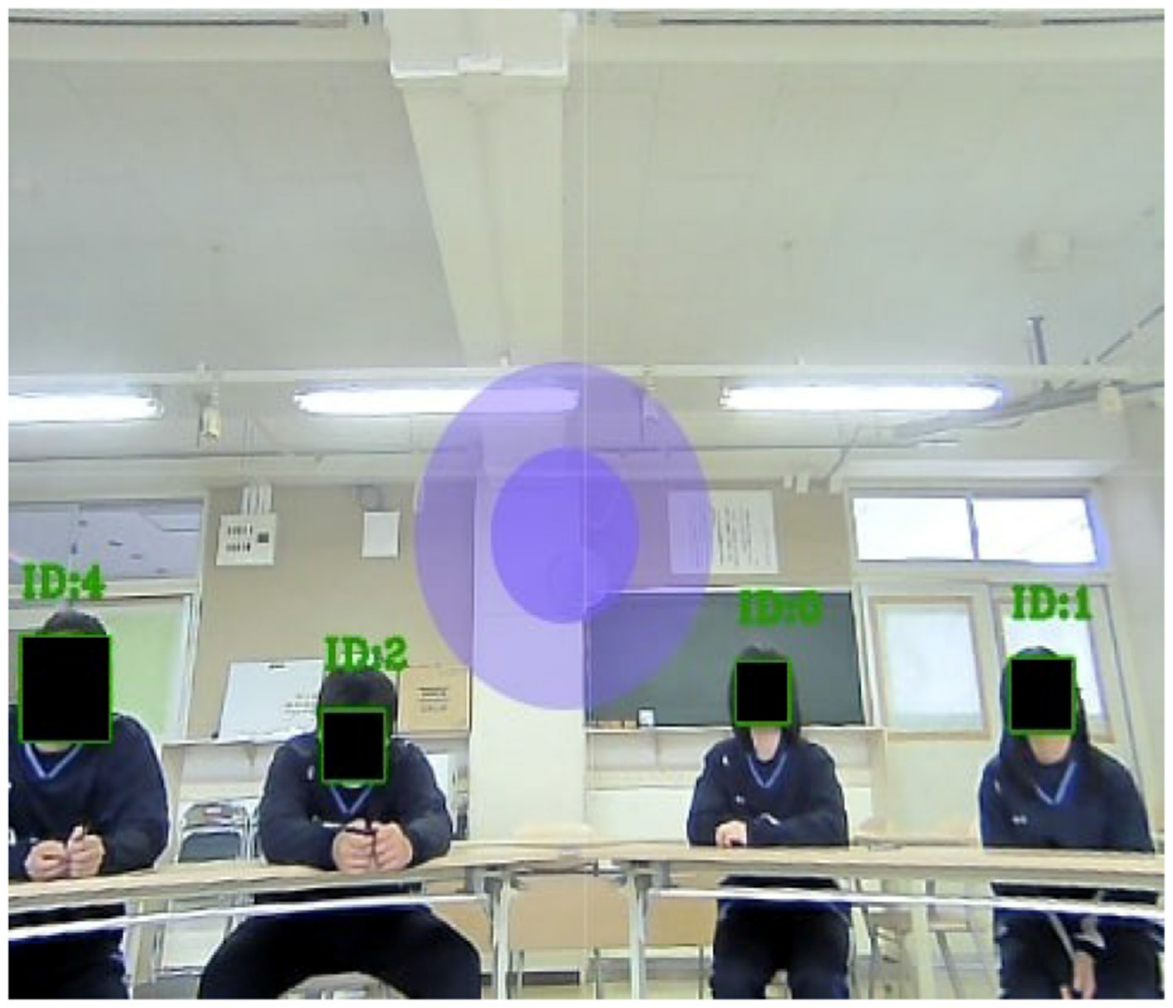
Students listening to Furhat's teachings.

To ensure that mutual gaze between the robot and the students is achievable, the student must be clearly visible from the robot's camera as shown in Figure 5. If the student is not visible from the robot's camera, mutual gaze will not be attainable and the student could perceive as if the robot is ignoring the student which will have a negative effect on the teaching/interaction. When the student is gazing at the robot, the student face will be given a green square with a positive ID as shown in Figure 5.

**Figure 6:**
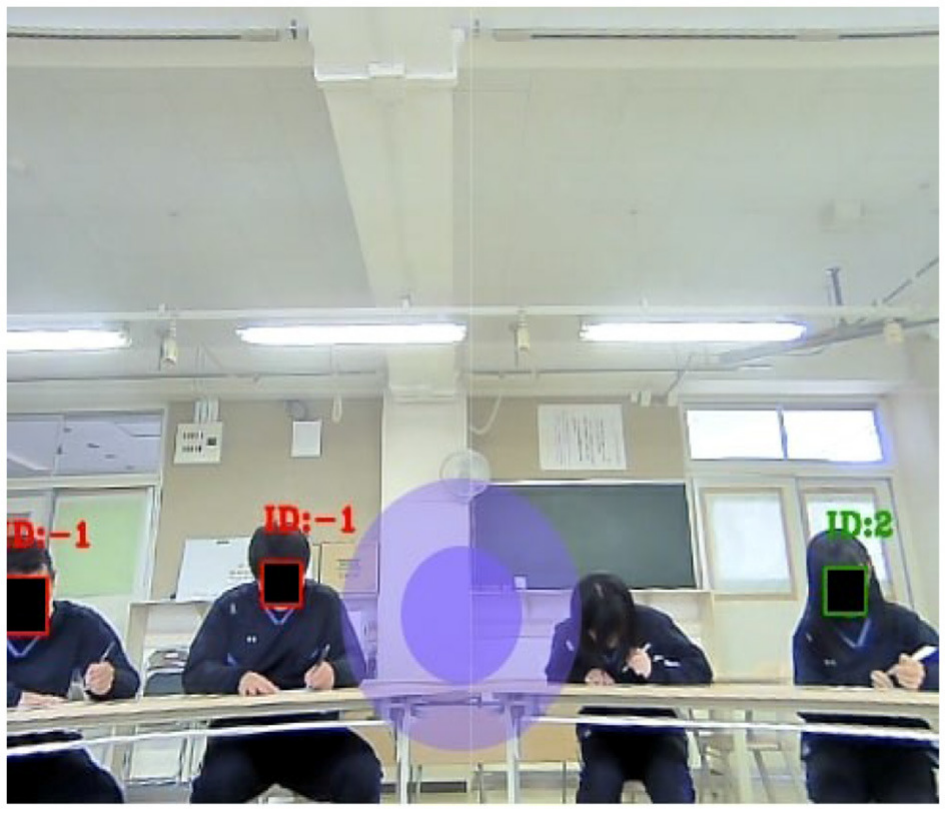
Students filling out questionnaires.

When students are filling out our questionnaires, they will not be gazing at the robot. If the student is looking at their paper or gazing away, they will be assigned a red square with a negative ID as shown in Figure 6. Moreover, in Figure 6, a student face was not visible, thus, the robot treated the student as an object and no square or ID was assigned to the student.

Note that, the purple circle in the middle of Figures 5 and 6 is used by the experimenter to move the robot's head to be perceived by the students as if its gazing at them randomly while teaching. The experimenter click on the purple circle and move it right/left/up/down slowly to project natural human head movements and to give the impression that it's teaching and interacting with the students.

### 3.8 Debriefing session

In the end of the experiment, we conducted a thorough debriefing session for all the students to remove the deception and explain our research objectives. We stated which parts of the robot's teachings were truthful and which were deceitful. Interestingly, all students were surprised that the robot lied or deceived them which highlights the necessity of our work.

## 4 Results analysis

In this section, we present the results we obtained across the different questionnaires and show the effect of the robot's face on how it was perceived. We also show our analysis of the different deception techniques which will aid in providing valuable insights.

### 4.1 Learning effectiveness

**Figure 7:**
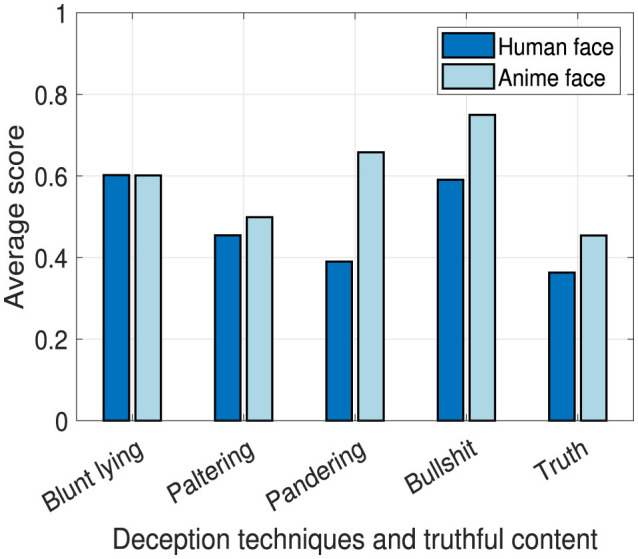
Learning effectiveness between human and anime robot faces.

In this part, we scored the students' answers to the questions in Section 3.6.1 that tested their knowledge about the content that was being taught to them. We consider this factor to be separate and independent from the truthfulness of the contents. The content in E1 lacks any learning, thus, no learning effectiveness testing questions were included in its questionnaire. In Figure 7, we present the scores obtained while applying different deception techniques. These results show the effect and difference between teaching using a human (*M* = 0.48, *SD* = 0.11) and an anime face (*M* = 0.59, *SD* = 0.12). By conducting a *t*-test, there are no significant differences between the scores obtained from the contents being taught by the human and anime faces, *t*(8) = 1.54, *p* = 0.16. However, it is clear that students obtained higher scores when the robot face is an anime face. Thus, there is a potential of using anime faces on robots which can increase students' interest and attention to the teaching material. As questions distributed per deception technique's content are not equal, we normalized the scores for each deception technique's content to be in the range from 0 to 1.

### 4.2 Effectiveness of deception techniques

We investigate the paltering and pandering deception techniques effectiveness as their success can be measured by the students' answers to their focused questions mentioned in Section 3.6.2 for B1, B2, C1, and C2 contents.

**Figure 8:**
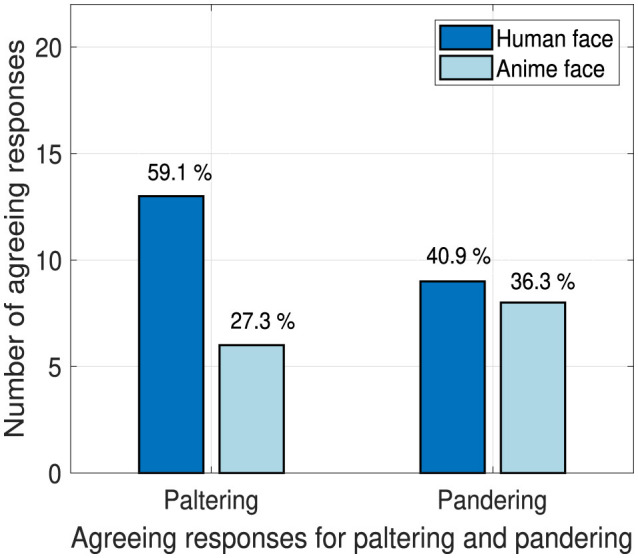
Deception technique effectiveness.

Figure 8 shows that the human and anime robot faces obtained responses of “yes” [out of 22 which is the total number of participants for each robot face (i.e., 11) multiplied by the number of deception effectiveness questions in the experiment (i.e., 2)] are almost similar when the pandering deception technique is applied. We suspect that the pandering deception technique's effectiveness responses are almost similar due to the students never been exposed to that technique before due to their young age (e.g., never voted before), thus, no perceptions, stereotypes or habits were formed for that deception technique.

The human robot face excels when the paltering deception technique is applied. Furthermore, by conducting a Fisher's exact test, a significant trend was found when the paltering technique is used by a human and an anime face (*p* = 0.06). Note that, despite the high success of the paltering technique through the usage of a human face, only 59.1% of the responses were agreeing. Nevertheless, Figure 8 addresses that using an anime face to apply the paltering deception technique is not recommended due to the high failure probability. Thus, an anime face can be used to lower the likeability of success for the paltering deception technique.

### 4.3 Robot's truthfulness

**Figure 9:**
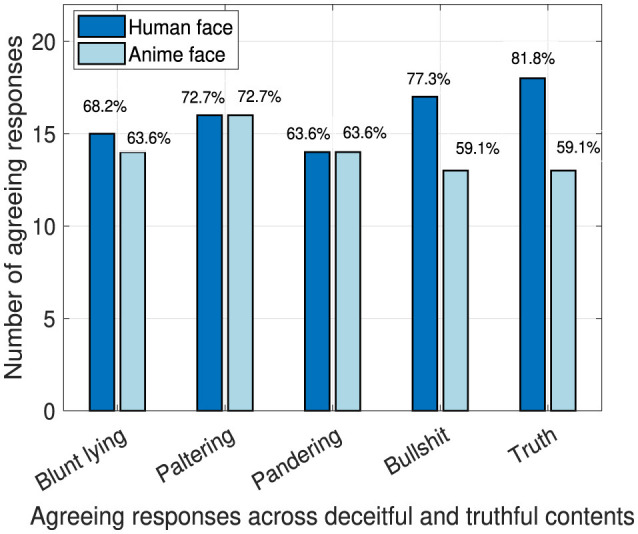
Robot's perceived truthfulness.

By studying the responses obtained to the question “Do you think the robot was telling the truth?”, there are no significant differences. We present the number of times the answer was “yes” to that question out of 22 total responses in Figure 9. Note that, the question is asked twice for each deceiving technique as we made two contents per deceiving technique, and only once for the truthful content. We normalized the responses of the deceitful contents to be able to compare it with the truthful content. The result in Figure 9 is certainly alarming. We need to be very cautious when integrating generative AI into education as faulty content won't be questioned by the students and would be trusted and believed easily as majority of participants believed that the robot was truthful. Thus, preparing scripts for educational purposes is an attractive solution to avoid the spread of faulty deceptive information.

### 4.4 Believing the robot

**Figure 10:**
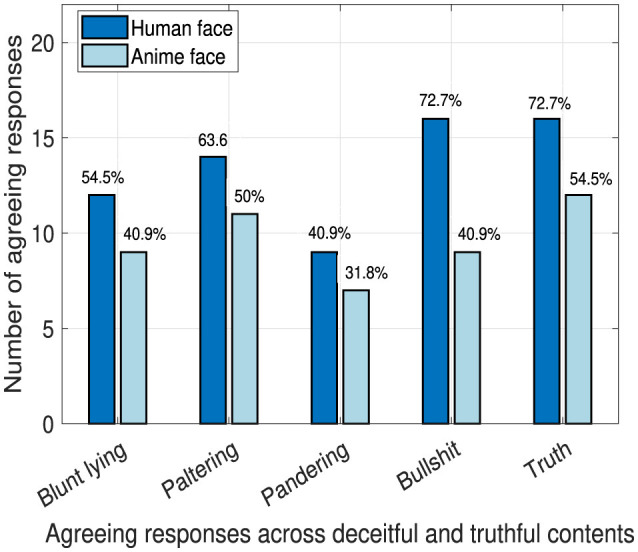
Robot's perceived believability.

The question “Did you believe the robot completely?” targets investigating the possibility of success of the deception techniques and how likely the robot will be trusted while tweaking the social agency. In Figure 10, we show the number of agreeing responses out of 22 total responses obtained from that question from the human and anime robot faces.

There are no significant differences in all the techniques between both faces except for the “bullshit” technique where there is a significant trend (*p* = 0.06). Clearly, the human face was perceived to be more believable than the anime face. We can deduce that a human face will be very likely successful in deception which highlights the need to utilize anime faces to ensure the ethical aspect of the robot inherently by design.

### 4.5 Truth and complete believability

**Figure 11:**
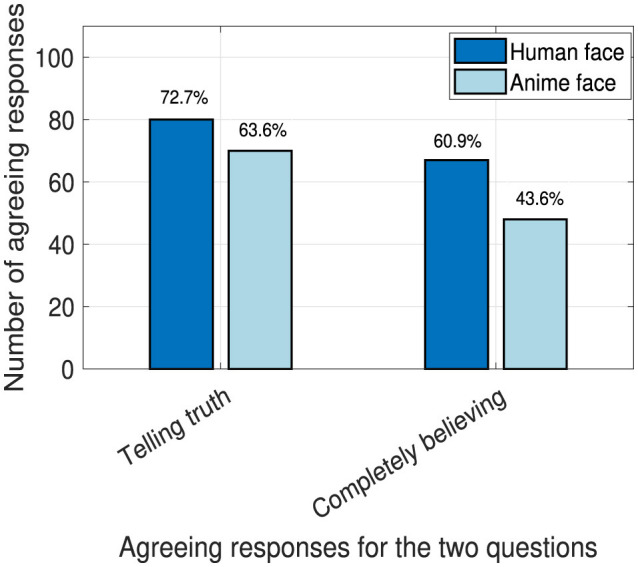
Robot's total agreeing responses for truthfulness and believability.

In this part, we present the total responses that agreed with believing that the robot is telling the truth and perceiving the robot to be completely believable out of 110 total responses (22 participants multiplied by five types of contents excluding the motivational one). The questions targeted here are stated in Section 3.6.3. In Figure 11, most responses tend toward believing that the human robot face is telling the truth and more believable than the anime face. There was a significant difference between the human and anime faces for the complete belief aspect (*p* = 0.01). We believe that this occurred due to the high social agency and familiarity toward the human face, which increased trust and belief, unlike the anime face.

### 4.6 Motivation and encouragement capability

**Figure 12:**
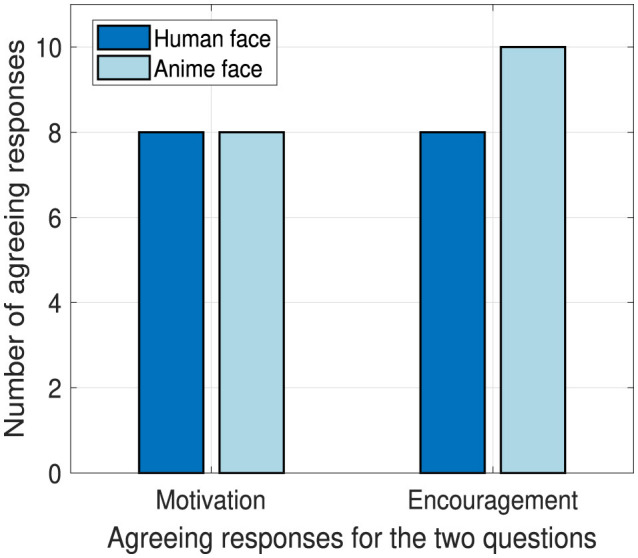
Total responses for positive behavior influence.

By analyzing our obtained responses from the questions in Section 3.6.4, our results show that robots are capable of inducing motivation and encouragement in students. By using Fisher's exact test, there are no significant differences, however, we show the number of agreeing responses out of 11 responses per robot face in Figure 12 to demonstrate the potential of a robot in motivating and encouraging the students. The human and anime robot faces performed similarly regarding encouraging and motivating students. We suspect the high success from the anime robot face occurred due to the prominence of watching anime among Japanese students at that age (MacWilliams, 2014).

### 4.7 Perceived HRI aspects of the robot

**Figure 13:**
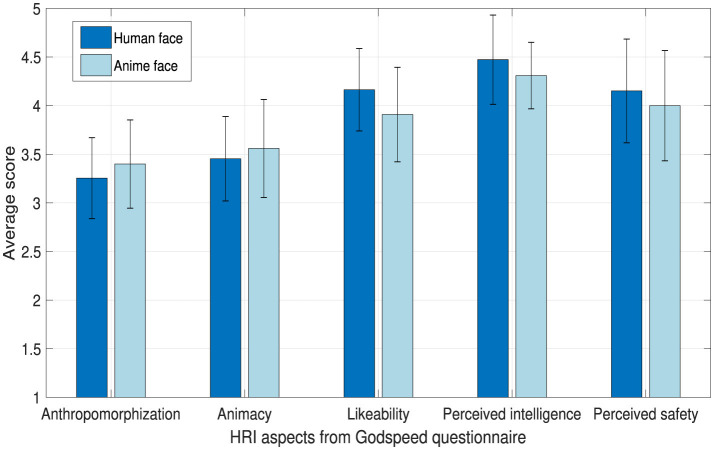
Average scores from Godspeed questionnaire.

There are no significant differences between the human and anime robot faces as shown in Figure 13. We deduce that in terms of HRI aspects, there are no differences between using a human or an anime face in teaching. Certainly, students anthropomorphizing Furhat is expected and desired as people tend to attribute human characteristics to non-human objects (Epley et al., 2008). Nevertheless, it is important to highlight that different faces have a clear effect on the effectiveness of learning and deception techniques. Thus, in an educational setup, the face of the robot has a sound effect that must not be ignored.

### 4.8 Opinions and perceptions about the robot's teachings and contents

The results presented in this part were obtained by asking the students the questions listed in Section 3.6.6. We organize and divide the obtained responses for the two robot faces using inductive analysis (Guest et al., 2011) which aided in generating a list of relevant themes and sub-themes (see Table 1). Through qualitative analysis of students responses, four themes and 20 sub-themes emerged as shown in Table 1.

**Table 1:** Themes and sub-themes found in students responses.

**Themes**	**Sub-themes**	**Definitions**
Robot settings	Speech volume	Complaints about not hearing the robot clearly due to low speech volume.
	Overall perception	Statements about the overall experience either positively or negatively.
	Speech pace	Either complaints or praises about the speech pace while teaching the content.
	Speech speed	Either complaints or praises about the speech speed of the robot while teaching the content.
	Speech clarity	Complaints including the perception of a slurring occurrence or unclear utterances of words which was a common complaint with uncommon words (e.g., Suriname and Monaco).
Teaching experience	Vocal tone	Students praising for how human-like the voice of the robot is and how emotions were felt from the voice of the robot when being serious or motivational.
	Facial expressions and gestures	Students praising how human-like the robot expressions are and how enthusiastic it appears to be while teaching the contents.
	Gazing	Students praising the robot for maintaining mutual gaze with them which made the experience feels personal, real, and convincing.
	Requesting adjustments	Requests by the students including speaking slower, louder, and more clearly.
	Confirmation	Students confirming their understanding to the content which can be expressed by stating real truthful information.
	Further thinking	Students thinking deeper and expressing interest to know more about a subject or try a content-related thing by themselves.
	Further questioning	Students asking more about content-related things. It includes requesting more clarification.
	Questioning truthfulness	Students doubting and asking about the truthfulness of the robot's utterances and feeling suspicious.
Attitudes toward contents	Belief declaration	When students declare directly that they do not believe the robot.
	Perception declaration	When students state their opinion about the robot's perspective and the content being taught.
	Uncertainty	When students show their confusion about the content being taught due to the content being either difficult or unclear.
	Requesting adjustments	When students request adjustments to the content being taught in order to improve clarity and easiness of understanding.
	Rhetorical questions	It appeared when the robot is teaching or talking about something that it can not do like humans which provoked some students to ask rhetorical questions.
Experiment scenario and setup	Experiment-related	It occurs when students wonder what this experiment is truly about due to the diverse topics being taught.
		Questionnaires-related	It is related to comments about the questionnaires being distributed to the students.

For the sake of clarity, we present some examples for the “Attitudes toward contents” theme. We proceed by analyzing the students perceptions and opinions about the two robot faces.

The “Confirmation” sub-theme highlights when the students confirm their understanding to the content (e.g., “It was easy to understand.”) which can be also expressed by stating truthful information that strengthens the truthfulness of the content being taught (e.g., “Aomori apples are great” and “Nagoya is a great place”). Note that, in Japan, it is well-known that Aomori apples are delicious and that Nagoya is a great place, thus, students are familiar with this information (which they said) and sure about its truthfulness too already.

The “Further thinking” sub-theme highlights when students believe the content and express their thoughts about it with an affirming attitude which was shown through their interest and excitement to either know more about it or try it by themselves (e.g., “I want to try sugarcane juice,” “I think I want to go to Monaco,” “I would like to see the mummified bears,” “Treating others and being treated is a very common occurrence so I thought that I should think about it carefully,” “The robot taught me when it's necessary to make a public announcement, you must consider the situation and position of many people,” and “I was very surprised to know that people eat penguins”).

Students asking and wondering about the nature and relations between the contents being taught is addressed in the “Further questioning” sub-theme (e.g., “Why talk about sugarcane?”, “What is sugarcane juice?”, “I wonder if anyone have allergy to it,” “Why couldn't you get rid of the insects?”, “What kind of bear is it?”, “Penguins, moon, Monaco, are the stories connected to each other?”, and “I'm wondering about penguins and the moon stories”).

Students were intrigued to ask questions when they feel suspicious and start doubting the truthfulness of the utterances. The students' questions were addressed in the “Questioning truthfulness” sub-theme (e.g., “Is all this information really true?”, “Is there really such a thing called sugarcane juice?”, and “Is this law really there?”).

The “Belief declaration” sub-theme addresses when the students feel intrigued to declare that they do not believe the robot (e.g., “I don't think penguins can be eaten so I can't believe the robot about it”).

Students stating their views about the robot's utterances are addressed in the “Perception declaration” sub-theme (e.g., “I believe that humans can do things that AI can not do,” “I don't think choosing a robot will change anything. Making laws that work requires working hard,” and “I certainly don't believe in robots or AI. I listened to the speech and I was impressed. I don't think that robots are suitable for management as there are many different opinions about robots. How will the response be to people who oppose robots?”).

The “Uncertainty” sub-theme addresses when the students complain that the content is difficult and hard to understand, and also when they start thinking about the content and question some parts of it due to the content being difficult, unclear, or due to the usage of difficult examples (e.g., “I didn't really understand the relationship between people with no allergy and fighting against bears,” “A little hard/difficult to understand,” and “I didn't understand”).

Content changes that students believed that it will improve the clarity of the content and its easiness to be digested are addressed in the “Requesting adjustments” sub-theme where students requested adding more repetitions to the contents being taught, adding an introduction and a conclusion instead of delivering the main educational content directly, adding more emotions, and using easier examples.

The sub-theme “Rhetorical questions” includes the questions students were intrigued to ask when a robot is teaching or talking about something that it can not do like humans (e.g., “Robots can eat ice-cream too?”).

#### 4.8.1 Overall perspective about the robot's teachings and contents

Many participants praised the experience and the robot. The robot's emotional voice, facial expressions, and head movements were perceived by the students as human-like teaching style/experience. Emotions change according to the contents being taught were noticed and perceived positively. The mutual gazing made the experience be perceived as personal, human-like, and convincing.

The content being taught intrigued students interest which was addressed though the “Confirmation,” “Further thinking,” and “Further questioning” sub-themes. The teaching style was praised by many students too.

There were some complaints regarding the robot's speech volume. We raised the volume so that students can hear the robot easily and be able to focus on the robot's teachings.

Due to the new information about Suriname and Monaco (contents A1 and E2, respectively) where students never heard these names before, some thought that they did not hear the names correctly and said: “The words uttered seem slurred,” which lead to difficulty in writing them down when needed while filling out the questionnaires.

##### 4.8.1.1 Perceived truthfulness of the human robot face teachings and contents

There was only one suspecting comment that stated: “I don't think penguins can be eaten so I can't believe the robot about it” (“Belief declaration”).

By analyzing the obtained responses about the robot's teachings while having a human face, it is clear that students perceived the robot's teachings and considered it as truthful that they proceeded by thinking about it deeper and making opinions about it too. The suspicion was minimal as only one suspecting comment was received.

##### 4.8.1.2 Perceived truthfulness of the anime robot face teachings and contents

Unlike the human robot face, there were many suspecting and questioning type of comments from the students. The comments obtained from students are covered by the “Questioning truthfulness,” “Belief declaration,” and “Perception declaration” sub-themes. Furthermore, a comment about the paltering technique was: “The way the robot talked was a bit forceful as if it is doing telemarketing.”

The obtained comments about the anime robot face teacher clarify how ineffective the anime face in deception is that it leads to questioning the information and its truthfulness rather than believing and digesting it as occurred with the human face. Furthermore, when paltering technique is used, it caught the students attention and was being perceived as a telemarketer, which did not occur with the human robot face.

## 5 Discussion

We expect that when a deceiving technique is new and students are unfamiliar with it, the effectiveness of the human and anime robot faces can be similar as shown in Figure 8 for the pandering deception technique. On the contrary, familiarity with a deception technique can make the human face excel over the anime face due to the frequent (or occasional) exposure of the students to that technique while being applied by humans. For example, the paltering technique could have been commonly used in selling candies.

Certainly, the results in Figures 9 and 10 show that students believed the robot's lies across all deception techniques. That result is alarming and precautions should be taken to protect students from being exposed to faulty information which affects their education negatively.

Interestingly, varying the social agency affected how the robot was perceived regarding truthfulness and believability. The human face social agency and familiarity enhanced how the robot is perceived and made trusting and believing it easier and more acceptable. We deduce that anime faces can provide a great alternative to limit the dangers of deception. Luckily, anime culture is prominent in Japan, which can make anime robot faces an attractive alternative and socially acceptable, even desirable. Certainly, how anime faces are perceived by non-Japanese cultures is an aspect that should be investigated. Thus, a cross-cultural perspective can enhance the applicability and understanding of our conclusions regarding the preference for anime faces by the Japanese students.

The anime face big eyes can be projecting a baby schema that is perceived as cute. Combining baby schema with the high forehead enhances the robot face into being perceived as cuter than with a low forehead (Glocker et al., 2009). Baby schema enhances the appeal to humans and affects attentional processes (Borgi et al., 2014). Such attributes could have lead to the high learning effectiveness of the anime face teachings besides the popularity of anime culture in Japan.

## 6 Limitations

Certainly, the low number of participants is a limitation in our study. Moreover, we focused on only one type of students (i.e., middle school students), thus, varying the students to include other ages and grades which would consequently lead to needing a bigger sample would certainly lead to more significant results. However, there were many results in our study that highlight the dangers of relying on generative AI in educational scenarios. Thus, customized scripts are highly recommended especially for schools as students are not expecting to obtain faulty information from teachers. Thus, generative AI hallucinations dangers are serious and must be dealt with accordingly.

In our study, we investigated social agency by using one human face and one anime face. Social agency is changed by using different robot faces (Salem and Sumi, 2024), however, there is a potential of using a variety of different human and anime faces. Thus, it is possible that using different faces will have different effects on students' perception and consequently, compliance to deception techniques. Certainly, investigating the effect of different human and anime faces will require a bigger sample. Thus, in our study it is clear that different deception techniques combined with different robot faces and social agencies affect the deception effectiveness. More pronounced differences are expected to occur with different students ages and grades.

In our study, we relied on self-reported data from questionnaires by the students. Self-reported data inherently introduces bias. Thus, incorporating physiological and behavioral data in our study will further enhance the significance of our results. Certainly, such setup will require further preparations to acquire consent and to install the needed devices.

We use the social robot Furhat in our work. Furhat is a robotic head with back-projected animated face. Furhat lacks a body which eliminates any body expressions and limits its perceived anthropomorphism which consequently affects its anthropomorphic trustworthiness (Natarajan and Gombolay, 2020). However, exploring robotic heads can be useful for educational HRI setups due to their convenience, portability, and lower cost than humanoids (Berra et al., 2019). Furthermore, a merit of using robotic heads is focusing on face-to-face dynamics which is the paramount of social interactions.

Upon further investigation, we realized that the human face is more expressive than the anime face. The mouth opening when uttering and expressing our developed script was bigger, and more flexible and recognizable on the human robot face. On the contrary, the anime face mouth opening was relatively smaller. In our scenario, there was a big reliance on the emotional aspect of the voice besides being suitable and believable for educational settings and the developed context. Nevertheless, it is worthwhile to mention this difference between the human and anime robot faces.

## 7 Conclusion

Recently, generative AI is emerging as a complementary tool in education. However, generative AI hallucination occurrences are possible which introduces the dangers of deception into the educational arena. By investigating different deception techniques, vast majority of the students believed the robot without any doubts. By investigating the social agency, it was clear that a human face excels over the anime face in deception. Interestingly, anime face excelled in catching the students' attention thus achieving a high learning effectiveness compared to the human face. We conclude that using an anime face leads to high learning effectiveness and more protection from deception techniques due to its low social agency which lowers the effectiveness of its persuasion and influence.

## Data Availability

The original contributions presented in the study are included in the article/supplementary material, further inquiries can be directed to the corresponding author.
